# Gender-Based Screening for Chlamydial Infection and Divergent Infection Trends in Men and Women

**DOI:** 10.1371/journal.pone.0089035

**Published:** 2014-02-19

**Authors:** Susan M. Rogers, Charles F. Turner, William C. Miller, Emily Erbelding, Elizabeth Eggleston, Sylvia Tan, Anthony Roman, Marcia Hobbs, James Chromy, Ravikiran Muvva, Laxminarayana Ganapathi

**Affiliations:** 1 Statistics and Epidemiology Division, Research Triangle Institute, Washington D.C., United States of America; 2 Queens College and the Graduate Center, City University of New York, Flushing, New York, United States of America; 3 School of Medicine, University of North Carolina, Chapel Hill, North Carolina, United States of America; 4 School of Medicine, Johns Hopkins University, Baltimore, Maryland, United States of America; 5 Center for Survey Research, University of Massachusetts at Boston, Boston, Massachusetts, United States of America; 6 Statistics and Epidemiology Division, Research Triangle Institute, Research Triangle Park, North Carolina, United States of America; 7 Baltimore City Department of Health, Bureau of STI/HIV Prevention, Baltimore, Maryland, United States of America; 8 Research Computing Division, Research Triangle Institute, Research Triangle Park, North Carolina, United States of America; Xavier Bichat Medical School, INSERM-CNRS - Université Paris Diderot, France

## Abstract

**Objectives:**

To assess the potential impact of chlamydial screening policy that recommends routine screening of women but not men.

**Methods:**

Population surveys of probability samples of Baltimore adults aged 18 to 35 years in 1997–1998 and 2006–2009 collected biospecimens to estimate trends in undiagnosed chlamydial infection. Survey estimates are compared to surveillance data on diagnosed chlamydial infections reported to the Health Department.

**Results:**

Prevalence of undiagnosed chlamydial infection among men increased from 1.6% to 4.0%, but it declined from 4.3% to 3.1% among women (p = 0.028 for test of interaction). The annual (average) number of diagnosed infections was substantially higher among women than men in both time periods and increased among both men and women. Undiagnosed infection prevalence was substantially higher among black than non-black adults (4.0% vs 1.2%, *p* = 0.042 in 1997–98 and 5.5% vs 0.7%, *p*<0.001 in 2006–09).

**Conclusion:**

Divergent trends in undiagnosed chlamydial infection by gender parallel divergent screening recommendations that encourage chlamydial testing for women but not for men.

## Introduction

Untreated *Chlamydia trachomatis* infection increases the risk of pelvic inflammatory disease, chronic pelvic pain, and ectopic pregnancy in women [1]. Complications in men are rare, but untreated infection can lead to subsequent infection and morbidity in their female sexual partners. Repeat chlamydial infections are common in women [2]–[3] increasing their risk of serious reproductive problems.

Both the U.S. Preventive Services Task Force (USPSTF) [4] and the Centers for Disease Control and Prevention (CDC) [5] recommend chlamydial screening for sexually active women under 25 years of age (the CDC recommendation includes women through age 25) plus older women who are thought to be at increased risk based on their previous sexually transmitted infection history or patterns of sexual activity. This recommendation follows from the USPSTF conclusion that there is good evidence that screening for chlamydial infection in women who are at increased risk can reduce the incidence of PID [Pelvic Inflammatory Disease]. Routine screening of men is not recommended.

The impact of these divergent screening recommendations is not well understood, but “asymptomatic, untreated infections in men provide a reservoir of infection that may make it difficult to improve health outcomes in women” [USPSTF, p.130]. While concluding that the direct benefit to men of screening was likely to be small, the USPSTF noted that “screening for chlamydial infection in men may be beneficial if it were to lead to a decreased incidence of chlamydial infection in women. The USPSTF did not, however, find evidence to support this outcome … and identified this as a critical gap in the evidence” [4].

Several epidemiological trends could be expected to arise from the current gender-based screening policy. First, one would expect the number of chlamydial infections *diagnosed and treated* to rise substantially for females but not for males. Second, the prevalence of *undiagnosed asymptomatic infections* among men should be largely unaffected given the lack of male screening. Finally, one might expect the prevalence of *undiagnosed infection* in females to decline over time, but the extent of that decline might be limited by the undiminished prevalence of infection among their male partners. Nonetheless the USPSTF recommended that public health efforts focus on increasing screening for women for whom the direct benefits were most certain.

In this paper, we present evidence of the potential impact of this policy over the period 1997–98 through 2006–2009 in Baltimore, MD. Chlamydial infection became a reportable STI in Maryland in 1994 [5] and Baltimore has one of the highest reported rates of infection among U.S. cities [6]. We report surveillance data of *diagnosed* infections reported to the Baltimore City Health Department (BCHD) in 1998 and 2006–09. We also report results of population surveys of *undiagnosed* chlamydial infection conducted in 1997–98 and 2006–09 among probability samples of young Baltimore adults (Baltimore STD and Behavior Survey (BSBS) and Monitoring STDs Survey Program (MSSP)) [7]–[8].

By comparing surveillance counts of diagnosed chlamydial infection with survey estimates of prevalent undiagnosed infection during the same time period we provide a concise account of the epidemiology of chlamydial infection in an urban population over time. This methodology provides evidence – albeit imperfect – for evaluation of the relative effectiveness in a single community of public health efforts to diagnose and treat chlamydial infection that focus exclusively on women.

## Methods

### Overview

This article reports chlamydia results from two population-based surveys fielded by this article’s primary authors. The first survey, the Baltimore STD and Behavior Survey (BSBS), was fielded in 1997–98. STI prevalence estimates from this survey of Baltimore adults ages 18 to 35 were published in 2002 in the *Journal of the American Medical Association* [7]. The second survey, the Monitoring STIs Survey Program (MSSP), was fielded in 2006–2009. Chlamydia results from the second survey of Baltimore residents ages 15 to 35 year olds were published in 2011 in *Sexually Transmitted Diseases* [8]. To assess changes in chlamydia prevalence over the period 1997–98 to 2006–09, the present article combines the BSBS and MSSP datasets and restricts this composite sample to Baltimore residents ages 18 to 35.

Additional discussion of research methods can be found in Text S1.

### Trends in Diagnosed Chlamydial Infection

Cases of diagnosed chlamydial infection among 18 to 35 year olds reported by physicians and laboratories to the BCHD were tabulated by gender and age in 1998 (reported previously) [7] and 2006 through 2009 (tabulated by R Muvva). For the survey period September 2006 through June 2009, reported cases were summed and an annual average case count (based on the 33-month survey period) was derived. Percentages for population case rates were calculated using 1998 Census estimates of the number of Baltimore residents aged 18 to 35 years as the denominator; for 2006–09 the average annual population size was used (see Text S2 and Text S3 and Table S1).

### Trends in Undiagnosed Chlamydial Infections

*Population Surveys: 1997–98 BSBS and 2006–09 MSSP.* Estimates of trends in the prevalence of *undiagnosed* chlamydial infections were obtained from our population surveys of probability samples of the Baltimore population in 1997–98 and 2006–09. These surveys were designed specifically to provide estimates of and trends in the population prevalence of sexually transmitted infections among young adults in Baltimore, Maryland. The Baltimore STD and Behavior Survey (BSBS) used in-person household interviews with urine-based testing for *C. trachomatis* to assess infection prevalence among 18 to 35 year olds between January 1997 and September 1998. Details of the BSBS sample design and survey execution were published previously^7^. The 2006–09 Monitoring STIs Survey Program (MSSP) used telephone audio computer-assisted self-interviewing (T-ACASI) technology [9]–[12] and biospecimen collection kits sent out and returned by US mail to diagnose chlamydial infection among Baltimore residents aged 15 to 35 years between September 2006 and June 2009. We restricted our analyses to ages 18 to 35 in both surveys.

Our decision to screen adults through the age of 35 in the 1997–98 BSBS was motivated by the lack of information on the population prevalence of chlamydial infections in the Baltimore population. Investigators thought it might be possible to detect hidden pockets of undiagnosed infection in unsuspected segments of the Baltimore population. The 2006–09 MSSP maintained a top age of 35 because that survey included testing for *Trichimonas vaginalis* which is prevalent in women ages 30 to 49 [13].

#### Sampling and sample weighting

The sample for the 1997–98 BSBS was drawn from households selected from the Baltimore Real Estate Property Registry. Two sample strata were oversampled: (1) black men and (2) adults living in predominantly white census tracts with high levels of reported sexually transmitted infections to enhance the precision of estimates for these subgroups.

For the 2006–09 MSSP, our target population was young adults residing in Baltimore households with landline telephones. Over the course of the MSSP survey (September 2006 through June 2009), we estimate that approximately 15% of Baltimore households did not have a landline telephone (see Text S4). Four strata were sampled probabilistically. The first three strata were sampled using commercially-available, regularly updated information on Baltimore households and included households with a landline telephone (1) that were believed to contain someone aged 15–35 years, (2) that were believed to have no one aged 15–35 years, and (3) whose residents were of unknown age. A fourth stratum was constructed by selecting all known landline telephone numbers in Baltimore and removing numbers that appeared on the commercial list to ensure that each telephone number was in one and only one stratum.

Sample weights were derived for both surveys to adjust for the unequal probabilities of selection based on the sample design and for specimen nonresponse. Details of sample weighting procedures are provided in Text S5).

#### Interview/specimen collection

Respondents in both surveys completed detailed computerized behavioral questionnaires. Upon interview completion, respondents provided a biospecimen for chlamydial testing (see Text S6). Specimens were tested using a ligase chain reaction assay (LCx, Abbott Laboratories) in the BSBS and transcription-mediated amplification (APTIMA Combo2, Gen-Probe, Inc) in MSSP. Both tests demonstrate high sensitivity and specificity when used as screening tests for chlamydial infection [14]. To minimize the possibility of false positive results within our non-clinical population, all positive specimens were retested [15]–[16]. Chlamydial infection was defined as a repeatedly positive test result.

### Ethics Statement

All participants in the 1997–98 BSBS were adults. Interviews and specimen collection were conducted in the household and participants provided written consent for both the survey data collection and for the urine collection and STD testing. Adult participants in the 2006–09 MSSP telephone survey provided verbal consent. Minors aged 15 to 17 years in the MSSP were also recruited, and they provided verbal consent with parental/guardian permission and minor assent for the telephone survey. Parents were informed that their child’s survey and STI test results were confidential and that they would not be shared with parents.

Verbal consent was used for the telephone survey portion of the MSSP because there is no face-to-face contact in a telephone survey. Verbal consent is the widely-accepted norm for obtaining consent in telephone surveys. (Further details of the MSSP survey consent procedures are provided in Text S7.) Written consent was obtained for the mailed STI specimen collection portion of the MSSP. Respondents who agreed to provide a specimen for STI testing were mailed a collection kit (a maximum of three days after the T-ACASI interview) with instructions and a written consent form. Only specimens submitted to the laboratory with a signed consent form were tested.

All study procedures - including the foregoing consent procedures – were approved by the Institutional Review Boards (IRBs) of the Research Triangle Institute (BSBS and MSSP), the University of North Carolina at Chapel Hill (MSSP), the University of Massachusetts at Boston (MSSP), and the Johns Hopkins Medical Institutions (BSBS and MSSP).

### Data Access

The data required to replicate our substantive analyses will be available to authorized researchers under a restricted data use agreement with the Inter-University Consortium for Political and Social Research (ICPSR) at the University of Michigan. Because of the sensitive nature of these data and the risk of deductive disclosure of respondents’ identity, researchers must agree to the ICPSR’s terms of use which require, in part, compliance with the repository’s access regulations and codes of scientific conduct.

### Statistical Analyses

Our analysis of *diagnosed* infections is derived from a census of all infections reported by laboratories and health care providers to the Health Department (see Text S2). Our statistical analyses of the estimated prevalence of *undiagnosed* infections use survey data from respondents who provided biospecimens for chlamydial infection testing. We tabulated frequencies of demographic characteristics to obtain descriptive profiles of the survey samples and to control for differences in sample composition across the two survey periods. Survey estimates of infection prevalence were derived using sample weights and tabulated by gender, age, and race/ethnicity. Tests of the equivalence of prevalence estimates among population subgroups across the two survey periods (2006–09 versus 1997–98) were derived using crude and adjusted odds ratios. Adjusted odds ratios were calculated using logistic regression models to adjust for the effects of gender, race/ethnicity, age, education, and marital status on chlamydial infection. All statistical analyses accounted for the complex survey design using the *svy* algorithms of Stata, versions 10–12 [17]–[18].

#### Assessment of impact of missing data

To assess the impact of biospecimen collection non-response on survey estimates of infection prevalence, we previously used logistic regression to model the likelihood that the respondents would test positive based on sociodemographic, behavioral, and health-related variables. Our BSBS analyses suggested that prevalence estimates were robust in the face of missing urine specimens [7]. This result is consistent with findings from the 1995 National Survey of Adolescent Males (NSAM) and the National Longitudinal Study of Adolescent Health (Add Health, Wave 3). Biospecimens were missing for 12% to 28% of participants in these surveys, but multiple imputation (NSAM) and sensitivity analyses (Add Health) suggest that the impact of this biospecimen nonresponse on prevalence estimates was not likely to be substantial [19]–[20].

For the present article, we carried out parallel analyses using both single imputation logistic models and multiple imputation models using chained equations (MICE, see Text S8). The synthetic estimate of chlamydia prevalence derived from our single imputation modeling for missing MSSP biospecimens was 3.58% which was nearly identical to the estimate (3.54%) derived from tested specimens alone. MICE models were restricted to the black population since there were few infections that could be used to estimate MICE models for the nonblack population (0 among nonblack females in 1997–98 and 3 in 2006–09; 4 among nonblack males in 1997–98 and 1 in 2006–09). We used the MICE imputation procedures of Stata v12 to impute the substantial number of missing chlamydial infection tests (n = 493 of 1,856 black respondents) plus a small number of missing observations for some demographic variables. Predictor variables used in MICE imputations included age, education, married (or not), number of sex partners in past year, gonorrhea and chlamydia diagnoses in past year, dysuria and discharge in past two (BSBS) or three (MSSP) months, male gender, time period (1997–98 vs. 2006–09), interaction of male-by-time period, interaction of gender-by time period, sample strata, and sample weight. Logit models were used for all imputations except education and number of sex partners in past year. Imputation of these variables used ordered logit models. The chained equation multiple imputation procedure generated 60 sets of imputed data after a burn-in period of 100 iterations. Our hypothesis testing using MICE data took account of the complex sample designs of our population surveys using Stata’s *svy* estimation command.

#### Sensitivity analyses of assay performance

Sensitivity analyses were conducted to assess the impact of diagnostic test performance on prevalence estimates using a range of plausible estimates for the sensitivity (low = 0.869, high = 0.94) and specificity (low = 0.992, high = 0.999) of the assays used in each survey [14]. Tabulations examined the combination of effects from low (sensitivity = 0.869, specificity = 0.992) to high estimates of test performance (sensitivity = 0.94, specificity = 0.999) for the overall population and gender and racial subgroups.

## Results

### Trends in Diagnosed Chlamydial Infections

**Figure 1:**
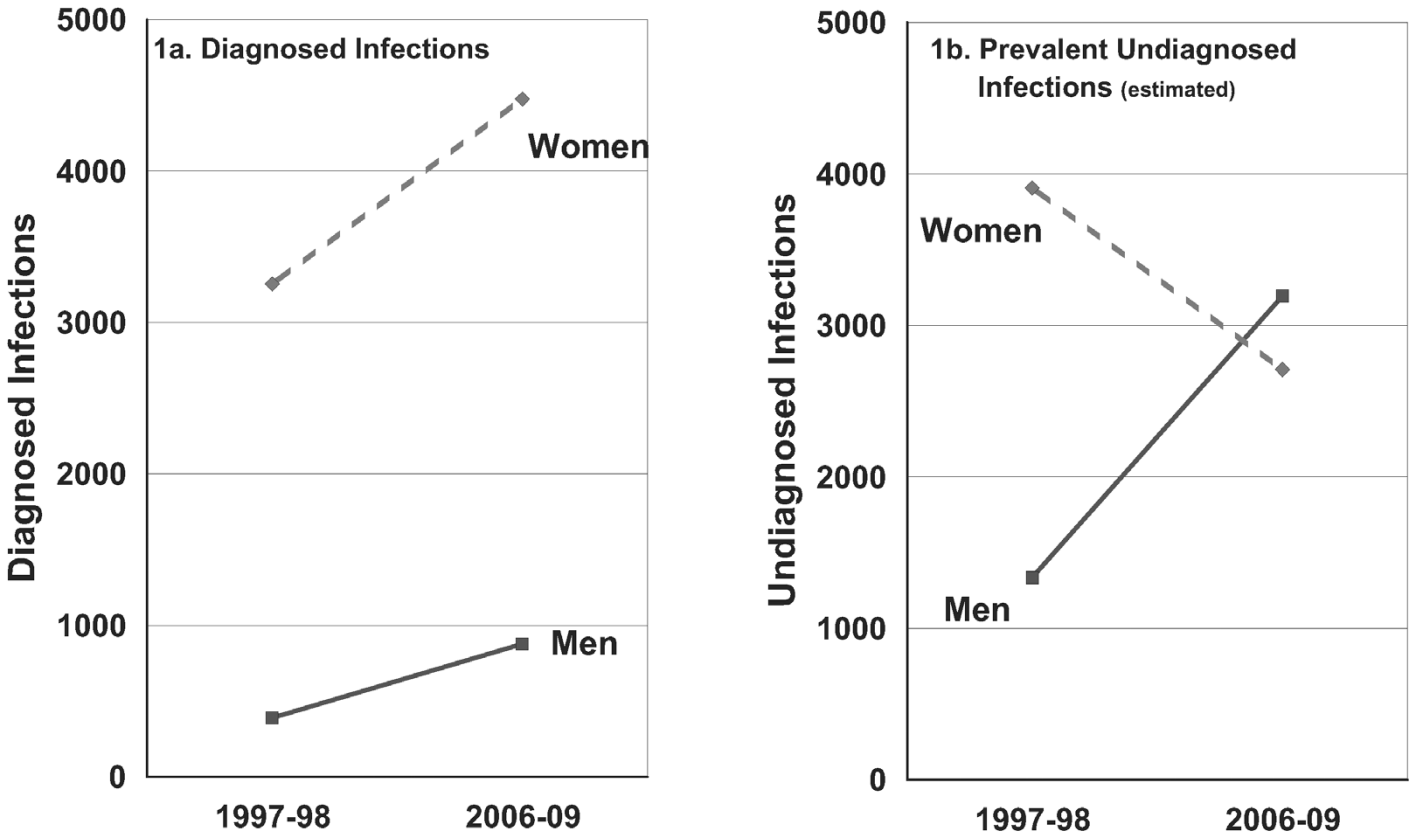
Reports of Diagnosed Chlamydial Infections (1a) and Estimates of Prevalent Undiagnosed Chlamydial Infections (1b) by Gender and Year among Baltimore adults, ages 18 to 35. See Text S2 for additional information.

Figure 1a provides the annual number of chlamydial infections ***diagnosed*** among Baltimore men and women ages 18 to 35. Among men, the number of diagnosed infections was 391 in 1998, and it increased to 878 per year in 2006–09. Annual diagnosed infections were substantially higher among women and the average annual number of infections diagnosed among women increased from 3,255 in 1998 to 4,475 in 2006–09.

### Trends in Undiagnosed Chlamydial Infections

#### Survey estimates of infection prevalence

Of the 3182 households selected for interview in the 1997–98 BSBS, 86% (2727) were successfully screened. 1224 eligible adults (aged 18 to 45 years) were identified and 1014 (82.8%) completed an interview. Of the 728 BSBS survey respondents aged 18 to 35 years, 579 (79.5%) provided a specimen adequate for testing.

In the 2006–09 MSSP, 73,318 telephone numbers were released over the survey period. 48,136 (65.6%) of these numbers were non-residential (out of service, businesses, faxes, etc.), 20,435 (27.9%) were residential, and the status of 4747 (6.5%) numbers was undetermined after repeated attempts. Of the residential numbers, 14,199 (69.5%) were screened and 4314 included one or more eligible household members aged 18 to 35 years. Interviews were completed with a randomly-selected 18 to 35 year-old from 2471 (57.3%) of those households; 1766 of these survey respondents (71.5%) mailed in specimens for testing.

Women were more likely to participate than men (as reflected by larger unweighted sample Ns, Table 1). Respondents in the 2006–09 MSSP were more likely to be non-Black, to report cohabiting with a partner, and to have graduated from college than respondents in the 1997–98 BSBS. While some of these differences are consistent with demographic change, e.g., college graduation rates among Baltimore women ages 18 to 34 years increased from 16.2% in 2000 to 27.8% in 2008 [20] other discrepancies may reflect the impact of differing survey methodologies. The impact of these differences cannot be precisely calibrated. Statistical controls are included to adjust for survey variation in sociodemographic composition across the two time periods. All adjusted ORs were calculated in logit regressions that included the following control variables: age in years, education, marital status. Adjusted ORs for comparisons within race categories included an additional control for gender. Adjusted ORs for comparisons within genders included an additional control for race (black vs. non-black). Calculation of the adjusted OR for the total population included controls for both gender and race.

**Table 1:** Sociodemographic characteristics of Baltimore survey respondents providing specimens for chlamydial testing in 1997–98 and 2006–2009.

	1997 – 1998 (a)			2006 – 2009 (b)			
Characteristic	Unweighted N	%	(95% CI)	Unweighted N	%	(95% CI)	P
Sample N	579	100.0	na	1766	100.0	na	
*Gender*							
Female	335	52.0	(46.7, 57.2)	1148	54.7	(51.7, 57.7)	0.378
Male	244	48.0	(42.8, 53.3)	618	45.3	(42.3, 48.3)	
*Race* (c)							
Black (not Hispanic)	316	65.4	(61.3, 69.3)	1046	58.4	(55.5, 61.2)	0.006
Non-Black (incl. Hispanic)	263	34.6	(30.8, 38.7)	719	41.6	(38.8, 44.5)	
*Age*							
18–19	48	10.3	(7.4, 14.1)	222	14.4	(12.4, 16.7)	0.059
20–24	138	24.9	(20.6, 29.6)	460	27.9	(25.3, 30.7)	
25–29	160	27.6	(23.2, 32.5)	501	26.6	(24.1, 29.3)	
30–35	233	37.2	(32.4, 42.4)	583	31.0	(28.4, 33.8)	
*Marital status*							
Married	123	21.1	(17.3, 25.5)	378	22.1	(19.8, 24.6)	<0.001
Cohabiting, not married	80	15.6	(12.2, 19.8)	375	24.7	(22.2, 27.4)	
Widow, divorced, separated	71	9.7	(7.2, 12.8)	61	3.0	(2.2, 4.1)	
Never married	305	53.6	(48.4, 58.7)	951	50.3	(47.3, 53.2)	
*Education*							
Less than high school	146	22.1	(18.4, 26.4)	280	17.0	(14.9, 19.4)	<0.001
High school graduate	183	36.6	(31.5, 42.1)	496	31.7	(28.9, 34.6)	
Some college/trade school	158	27.8	(23.4, 32.6)	475	26.5	(24.0, 29.2)	
College graduate	90	13.5	(10.6, 17.0)	514	24.8	(22.5, 27.3)	

Notes: Results for adults ages 18 to 35 from the Baltimore STD and Behavior Survey (BSBS) and the Monitoring STIs Survey Program (MSSP). Table shows unweighted base Ns for percentages and weighted estimates of the percentage of the survey respondents with sociodemographic characteristic. Weighted estimates account for differing probabilities of selection and post-stratification adjustment to match Census marginal for Baltimore, Maryland. 95% confidence intervals (CI) were calculated using statistical algorithms that take account of the complex sample designs of the surveys. Table excludes all respondents who were missing a chlamydia test result.

(a) The BSBS survey was conducted between January 1997 and September 1998.

(b) The MSSP survey was conducted between September 2006 and June 2009. Mailed urine specimens were received by the laboratory through August 2009.

(c) Persons who identified themselves as Hispanic are classified as Non-Black for both BSBS and MSSP analyses. This differs from our previous analysis of the BSBS in which persons were assigned to their chosen racial category; Hispanic origin was ignored. Of the 20 BSBS respondents who claimed Hispanic origin, 11 identified their race as “Other” and 2 selected “American Indian or Alaskan Native” as their racial category.

### Trends in Overall Prevalence

The estimated prevalence of undiagnosed chlamydial infection rose insignificantly from 3.0% (95% CI 1.7, 5.2) in 1997–98 to 3.5% (95% CI 2.6, 4.8) in 2006–09 (adjusted OR = 1.09, *p*>0.5; Table 2). Estimated prevalence was significantly higher among women (4.3%, 95% CI 2.2, 8.4) than men (1.6%, 95% CI 0.7, 3.4) in 1997–98 (*p* = 0.047). By 2006–09 the estimated prevalence was lower among women than among men (3.1% versus 4.0%), but the difference was not statistically significant (*p* = 0.414). In both survey periods, the prevalence of infection was substantially higher among non-Hispanic blacks than among other adults (4.0% vs. 1.2%, *p* = 0.042 in 1997–98 and 5.5% vs. 0.7%, *p<*0.001 in 2006–09).

**Table 2:** Trends in estimated prevalence of chlamydial infections by gender and race: Baltimore, 1997–98 and 2006–09.

	1997–98			2006–09			Odds Ratio (2006–09 vs. 1997–98)					
Population	Unweighted N	%	(95% CI)	Unweighted N	%	(95% CI)	Crude	(95% CI)	P	Adjusted	(95% CI)	P
Adults ages 18–35	579	3.0	(1.7, 5.2)	1,766	3.5	(2.6, 4.8)	1.19	(0.6, 2.3)	>0.5	1.09	(0.5, 2.2)	>0.5
*By Gender*												
Men	244	1.6	(0.7, 3.4)	618	4.0	(2.5, 6.6)	2.63	(1.0, 6.8)	0.05	2.46	(0.9, 6.6)	0.08
Women	335	4.3	(2.2, 8.4)	1,148	3.1	(2.2, 4.5)	0.72	(0.3, 1.6)	0.42	0.65	(0.3, 1.6)	0.33
*p* (gender comparisons)		p = 0.047			p = 0.414							
*By Race*												
Black (not Hispanic)	316	4.0	(2.1, 7.3)	1,046	5.5	(4.0, 7.6)	1.43	(0.7, 2.9)	0.34	1.12	(0.5, 2.4)	>0.5
Non-Black (incl. Hispanics)	263	1.2	(0.4, 3.4)	719	0.7	(0.2, 2.2)	0.60	(0.1, 2.8)	>0.5	(b)	(b)	(b)
*p* (race comparisons)		p = 0.042			p<0.001							
*By Gender and Race*												
Black Women	190	6.4	(3.2, 12.4)	720	4.5	(3.1, 6.6)	0.69	(0.3, 1.6)	0.38	0.59	(0.2, 1.4)	0.23
Black Men	126	1.1	(0.3, 3.6)	326	7.0	(4.2, 11.4)	6.70	(1.8, 24.6)	0.01	5.00	(1.3, 18.6)	0.02
Non-Black Women	145	0.0	na	427	0.8	(0.2, 2.8)	(a)	(a)	(a)	(a)	(a)	(a)
Non-Black Men	118	2.4	(0.8, 6.7)	292	0.7	(0.1, 4.5)	0.27	(0.03, 2.6)	0.26	(c)	(c)	(c)
*p* (gender-race comparisons)		p = 0.006			p<0.001							

Notes: Results for adults ages 18 to 35 from the Baltimore STD and Behavior Survey (BSBS) and the Monitoring STIs Survey Program (MSSP). Table shows unweighted base Ns for percentages and weighted estimates of the percentage of the population that was infected. 95% confidence intervals were calculated using statistical algorithms that take account of the complex sample designs of the surveys. ORs contrast the estimated prevalence in 2006–09 to the estimated prevalence in 1997–98. All adjusted ORs (AOR) were calculated in logit regressions that included the following control variables: age in years, education (4 categories: less than high school; high school graduate; some college; college graduate or higher); marital status (3 categories: married; cohabiting but not married; and single, separated, divorced, or widowed). AOR for comparisons within race categories included an additional control for gender (male vs. female). AOR for comparisons within gender categories included an additional control for race (black vs. non-black). AOR for comparison of the total population included controls for both gender and race.

(a) ORs cannot be calculated because no infections were found among nonblack females in 1997–98.

(b) AOR not shown. Category 4 of education (college graduate or higher) predicts non-infection perfectly. If observations with level 4 of education were excluded, AOR would be 0.69 (95% CI: 0.1, 3.7); p>0.5.

(c) AOR not shown. Categories 3 and 4 of education (some college, and college graduate or higher) predict non-infection perfectly. If observations with levels 3 and 4 of education were excluded, AOR would be 0.41 (95% CI: 0.04, 4.13); p = 0.45.

#### Sensitivity analyses of potential impact of test performance

Assuming a range of plausible values for diagnostic assay sensitivity and specificity, our sensitivity analyses indicate that the overall prevalence would range from 2.4% to 3.3% in the BSBS and from 2.9% to 3.8% in the MSSP. These estimates compare favorably with our population prevalence estimates.

### Variation in Estimated Trends by Gender and Race

Trends in the estimated prevalence of undiagnosed chlamydial infection varied substantially for men and women (Figure 1b and Table 2). Among men, estimated prevalence increased from 1.6% to 4.0% between 1997–98 and 2006–09 (adjusted OR = 2.5, 95% CI 0.9, 6.6; p = 0.08) whereas among women a decline in prevalence from 4.3% to 3.1% was observed over the same time period (adjusted OR = 0.7, 95% CI 0.3, 1.6; p = 0.33). An interaction test for non-equivalence of the estimated trends by gender was statistically significant (*p* = 0.028, test incorporates adjustment for differences in sociodemographic composition of samples).

We explored the possibility that the observed increase in infection prevalence among males in 2006–09 might be related to an increase in asymptomatic infection among men who have sex with men (MSM). Further analyses of the MSSP data indicate that this is not the case (see Text S9).

The divergence in trends by gender was particularly striking for black adults. Seven percent (95% CI = 4.2, 11.4) of black men had an undiagnosed chlamydial infection in 2006–09 compared to 1.1% (95% CI = 0.3, 3.6) in 1997–98 (adjusted OR = 5.0, 95% CI 1.3, 18.6, *p* = 0.02). In contrast, among black women, the prevalence of infection was insignificantly lower in 2006–09 than in 1997–98 (adjusted OR = 0.6, 95% CI 0.2, 1.4, *p* = 0.23). An interaction test for non-equivalence of the estimated trends in prevalence of undiagnosed CT infection among black respondents by gender was statistically significant (*p = *0.005). Parallel inferences are obtained when analyses use MICE procedures to impute missing observations (see Text S8 and Table S2). Using MICE-imputed data, we find a statistically significant (p = 0.049) increase in the estimated prevalence of undiagnosed chlamydial infection among black males from 1.6% (se = 1.6%) to 7.2% (se = 1.8%) while there is no significant change (*p* = 0.49) in estimated prevalence among black females. A test of the *gender by time period by prevalence* interaction using MICE data (*p* = 0.034) suggests that trends over the time period 1997–98 to 2006–09 in infection prevalence were significantly different for black males and black females.

### Variation in Estimated Trends by Age

**Figure 2:**
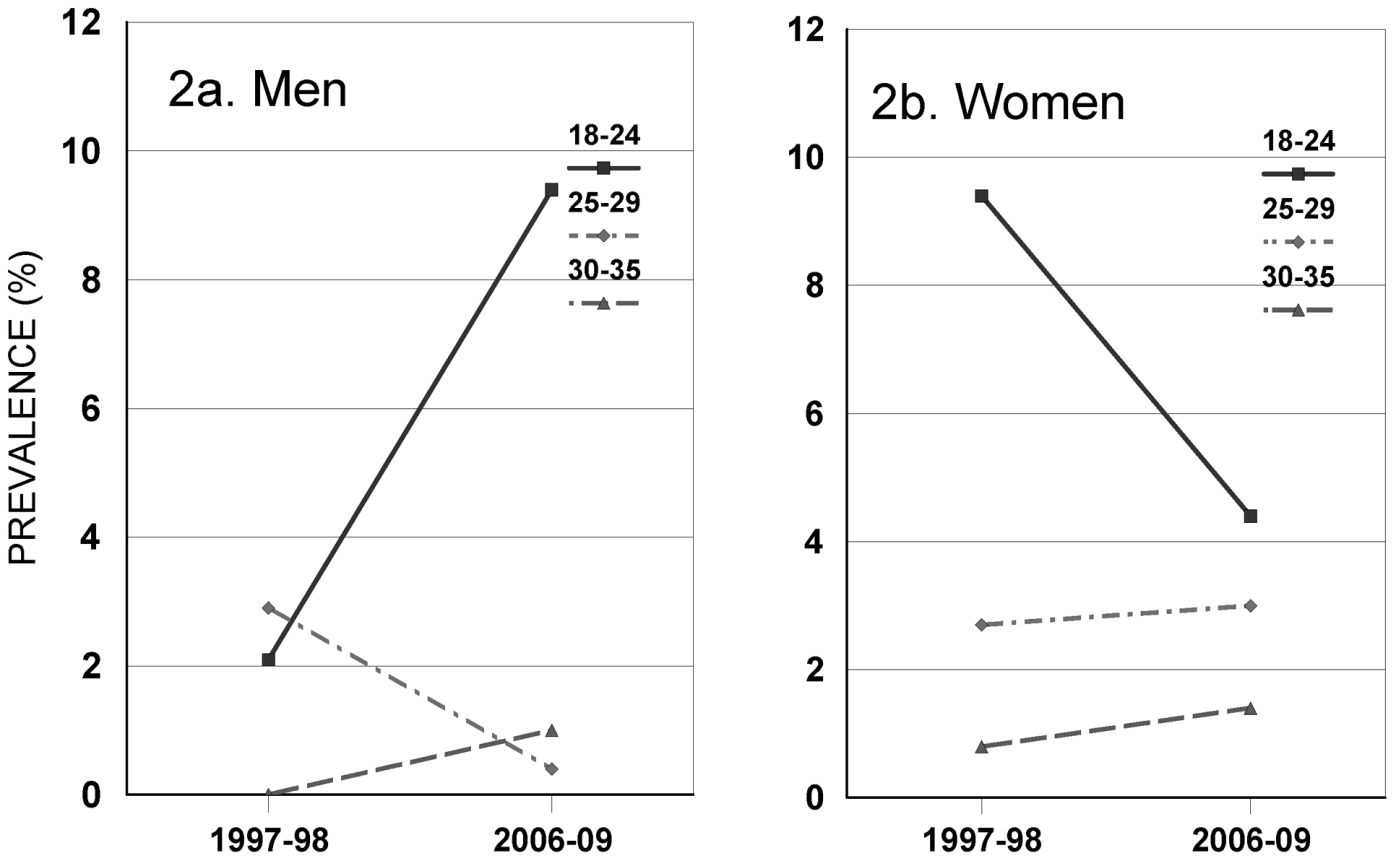
Estimated prevalence of undiagnosed chlamydial infection by age, survey year, and gender. (Source: 1997–98 Baltimore STD and Behavior Survey and 2006–09 Monitoring STIs Survey Program).

Among both women and men, the estimated prevalence of undiagnosed chlamydial infections was significantly lower among older adults (see Text S10 and Figure S1). As shown in Figures 2a and 2b, trends over time in infection prevalence varied by gender and age group. Figure 2b suggests that the prevalence of undiagnosed infection declined for the group for whom routine chlamydial screening is recommended –18–24 year old females. Estimated prevalence for this group declined from 9.4% (CI: 4.2–19.9) in 1997–98 to 4.4% (CI: 2.7–7.1) in 2006–09. However, this trend over time is not statistically significant (OR = 0.44, p = 0.11). In contrast, the estimated prevalence of undiagnosed chlamydial infections among 18 to 24 year old males (Figure 2a) trends in the opposite direction. It rises from 2.1% (CI: 0.7–6.0) in 1997–98 to 9.4% (CI: 5.6–15.4 in 2006–09). This increase in estimated prevalence is statistically significant (OR = 4.9, *p* = 0.01). Additional details of the analyses of age and infection prevalence are shown in Text S10 and Table S3).

## Discussion

Over the period 1997–98 to 2006–09, the overall burden of *diagnosed* chlamydial infection increased in Baltimore, primarily due to increased reports of diagnosed infection among women. The annual number of diagnosed chlamydial infections in women substantially exceeded that for men in each time period. While our population estimates suggest that the prevalence of undiagnosed infection among women remained relatively stable, substantial and unexpected increases in undiagnosed infections were observed among men, particularly young men less than 25 years of age. The divergence between men and women in these results is most pronounced among young black adults.

These results may be a direct consequence of current screening policies that encourage chlamydial testing and treatment among sexually active young females but not males. While current screening policy may have enhanced reporting of diagnosed infections and stabilized or slightly reduced the prevalence of undiagnosed infection among women, a growing reservoir of infection persists among young men in Baltimore. We suspect that reducing chlamydial infection and its associated morbidity may require policies that drain this reservoir of asymptomatic infection in the population from which most young women acquire their sexual partners, i.e., young men. These policies might include aggressive outreach to and treatment of recent sexual partners including presumptive treatment, repeat screening of positive cases following treatment, and community-based screening programs in high-prevalence locales.

A positive impact of screening on the prevalence of chlamydial infection has been difficult to demonstrate with confidence at the national and state levels. Reported cases reflect a mixture of incident infections treated due to symptoms and asymptomatic cases treated secondary to screening. Greater screening leads to more infections identified, and rates may actually increase over the short-term. At the national level, the prevalence of undiagnosed chlamydial infection appears to have decreased based on population data from NHANES [22] and the National Job Training Program [23]. In NHANES, the prevalence reportedly decreased by 53% in men and 31% in women aged 14–39 years over the period 1999–2008, however decreases in women aged 14 to 25 years, the age population targeted for annual screening, were not observed (19% overall reduction, 95% CI, −57, 57, *p* = 0.54) nor did overall prevalence among non-Hispanic black men and women change across the survey periods (0% overall reduction, 95% CI −37, 52). It is possible that statistical power was limited to reliably detect effects across time among subpopulations due to low disease prevalence and small sample sizes [24]. Nonetheless, national estimates, in conjunction with more precise estimates from well-designed and well-populated local probability samples, can be particularly informative for evaluating screening policy.

Chlamydia epidemics and their control are inherently local phenomena. The formation of sexual partnerships *benefits from* and sex itself *requires* geographic propinquity. Thus sexual networks tend to be geographically constrained, and the number and types of sexual partners available in different locales – as well as the prevalence of undiagnosed infection – may vary substantially across locales. Past research mapping the geography of sexual partnerships in Baltimore has demonstrated that there is a strong tendency for both STD clinic patients [25] and adults [26] in general to form sexual partnerships that are spatially assortative (See Text S11).

The ubiquity and intensity of local screening and treatment programs may also vary substantially reflecting variation in explicit policies, Health Department resources, zeal of staff and policymakers, and the perceived “seriousness” of STI problems. Because of variation in these factors, the benefits and cost-effectiveness of screening are more likely to be directly evidenced within a specific locale rather than nationwide. Our results, focused on the city of Baltimore, provide important evidence suggesting the limited impact of screening in that city. We believe that repetition of this research in other locales is warranted.

The inadequacy of screening strategies that focus exclusively on females is illustrated by studies that have intensively monitored young women. Batteiger and colleagues [2] followed 210 adolescent females treated for chlamydial infection for 3 years. Despite screening and assessments at 3-month intervals, 121 women had subsequent chlamydial infections following treatment. Based on genotyping results, 84% of these repeat infections were attributable to reinfection. Without screening and treatment of male partners, even an intensive regimen of chlamydial screening and treatment for women yielded disappointing results.

The availability of highly sensitive and specific urine-based nucleic acid amplification tests has facilitated the detection and treatment of asymptomatic infections among men. Public health authorities should consider routine screening of accessible populations of young men in locales in which incidence data indicate elevated rates of chlamydial infection among females. In addition to screening, strategies for effective control of chlamydial infection should include re-testing after treatment to guard against reinfection, aggressive case management, and evaluation and treatment of sex partners [27]. Based on mathematical models of chlamydia transmission, screening of men in addition to women [28] or expanded partner notification [29] substantially enhance the effectiveness of chlamydial screening on population prevalence.

While routine assessments of trends in the prevalence of undiagnosed chlamydial infections in the general population would be challenging, much could be learned from pilot programs focusing on accessible subpopulations, e.g., screening secondary school students in locales with high rates of incident STIs. In addition, we would recommend rigorous evaluations of the impact of expedited partner therapy, and active follow-up of patients whose partner treatment history cannot be verified [30].

### Limitations of Research

Our results should be interpreted with awareness of potential limitations to our inferences about trends in chlamydia prevalence. First, the 1997–98 BSBS study relied upon a ligase chain reaction assay whereas the 2006–09 MSSP study used a transcription mediated amplification system. The performance characteristics of these NAAT assays are comparable and very high, and our repeat testing of positive specimens should have theoretically increased test specificity without affecting test sensitivity. It is not possible, however, to dispel all uncertainty about the unavoidable non-equivalence of methodologies. (Manufacture of the ligase chain reaction assay was discontinued in 2003.).

Differences in survey methodologies (in-person vs. telephone interviewing) may also have introduced non-equivalence in sample composition. Since prevalence estimates are derived from biospecimen testing, the difference in questionnaire administration is not a major concern. However, the two methodologies had varying recruitment rates (82.8% in the 1997–98 in-person survey versus 58.7% in the 2006–09 telephone survey). While use of poststratification weights aligns our survey samples to the demographic distribution of the population, neither they nor covariate adjustments employed in our statistical analyses guarantee sample equivalence.

We also note that the sample size for our 1997–98 survey, 335 females and 244 males, was small although statistical hypothesis testing takes account of sample size. Larger sample sizes or a multi-year randomized controlled trial of alternative screening policies would certainly have produced much stronger evidence, however these approaches were not possible given NIH funding guidelines. Finally, we note that our results are restricted to one urban community, Baltimore, Maryland with a relatively high rate of (diagnosed) chlamydial infections (see Text S12). Whether these results may be replicated in other, similar urban settings is an important question for future research.

Nonetheless, we believe that the statistically significant divergence in infection trends by gender should be relatively robust to the possible effects of sample non-equivalence arising from methodological differences. While it is relatively easy to imagine scenarios in which trends over time could arise solely from variations in survey methodology, it is much harder to imagine conditions that would produce a significant gender interaction in which the prevalence of undiagnosed infection rises for men but declines for women.

### Strength of Evidence

The key question – for which the evidence presented in this article is suggestive but not determinative – is: Do estimated changes over time represent actual changes in the population prevalence of undiagnosed infections or could they arise as consequences of methodological variation in study procedures over time? We have detailed above the divergences in laboratory assays and in survey data collection that introduce methodological uncertainty into our inferences about changes over time in the prevalence of undiagnosed chlamydial infections. We employed a variety of procedures to reduce this inferential uncertainty (e.g., covariate controls for shifts in sociodemographic composition, extensive multiple imputation modeling to assess the impact of missing data, etc. Nonetheless, no such procedure can provide inferential certainty.

#### Why did prevalence “increase” among men?

Some readers of preliminary versions of this article wondered what produced the “increase” in the prevalence of undiagnosed infections among men. We believe a response to this question has at least three parts. First, it should be noted that the “increase” in infection prevalence over the observed time period for males was **not** statistically significant (*p* = 0.08). What was significant was the *divergence* in estimated trends among males and females. Second, our survey samples did not involve a static comparison of the same population cohort. Cohort variation in respondent characteristics and in initial infection prevalences (prior to reaching eligibility age for these surveys) can produce a variety of outcomes. Finally, given the observational nature of our data, it is not possible to answer with certainty questions about causality.

### Concluding Remarks

Overall our results demonstrate the importance of monitoring not only diagnosed chlamydial infection but also prevalent infection that persists, largely asymptomatic and undiagnosed, in a population. Women account for a disproportionate percentage of all newly acquired and diagnosed chlamydial infections in Baltimore. The relatively stable prevalence of undiagnosed infection among young women combined with an increase in the number of infections diagnosed over the period 1997–98 to 2006–09 suggest that current screening practices are having only a modest impact in reducing overall levels of infection among women. A corresponding small increase in the incidence of diagnosed infection and a large increase – of borderline statistical significance – in the prevalence of undiagnosed infections among young men suggest, however, that the burden of disease among men has not been substantially mitigated by current screening policies that focus exclusively on women. The potential consequences of these trends on the future incidence of infection within this population are troubling for both women and men.

We note that our results highlight the inability of surveillance statistics on the incidence of reported STI cases to provide an adequate picture of STI epidemiology. As the Institute of Medicine [31] and other commentators have concluded, tracking hidden epidemics of asymptomatic infections such as chlamydia requires new approaches to public health surveillance. Repeated population-based surveys using NAAT testing of self-collected urine (or other biospecimens) provide a feasible way to study trends in the prevalence of asymptomatic infections. These probability surveys, coupled with analysis of reports of treated infections, provide a new and much-needed tool for monitoring the total burden of STIs in the population.

## Supporting Information

Figure S1 **Estimated prevalence of undiagnosed chlamydial infections by age group and gender calcuated from combined 1997–98 and 2006–09 surveys.**
(TIF)

Table S1 **Counts of chlamydia cases among 18 to 35 year old adults reported to Baltimore City Health Department (BCHD) by gender and year, 1998 to 2009; together with estimated size of population ages 18 to 35 and calculated rate (Cases divided by Population).**
(DOCX)

Table S2 **Estimates for black respondents of gender-by-time (1997–98 vs. 2006–09) interaction using observed survey data on chlamydia prevalence plus imputations for missing data obtained by multiple imputation using chained equations (MICE) procedure.**
(DOCX)

Table S3 **Trends in estimated prevalence of undiagnosed chlamydial infections among Baltimore adults by gender and age group, 1997–98 and 2006**–09.
(DOCX)

Text S1 (DOC)

Text S2 (DOC)

Text S3 (DOC)

Text S4 (DOC)

Text S5 (DOC)

Text S6 (DOC)

Text S7 (DOC)

Text S8 (DOC)

Text S9 (DOC)

Text S10 (DOC)

Text S11 (DOC)

Text S12 (DOC)
