# Correction: Gender-Based Screening for Chlamydial Infection and Divergent Infection Trends in Men and Women

**DOI:** 10.1371/journal.pone.0099374

**Published:** 2014-05-28

**Authors:** The PLOS ONE Staff

Text S13 is omitted from the list of Supporting Information. It can be viewed below.

## Supporting Information

Text S13 (DOCX)
