# Supplementary figures and images for: Gender-Based Screening for Chlamydial Infection and Divergent Infection Trends in Men and Women
Source: PLoS One. 2014 Feb 19;9(2):e89035. doi: 10.1371/journal.pone.0089035 (PMC3929759; doi:10.1371/journal.pone.0089035)

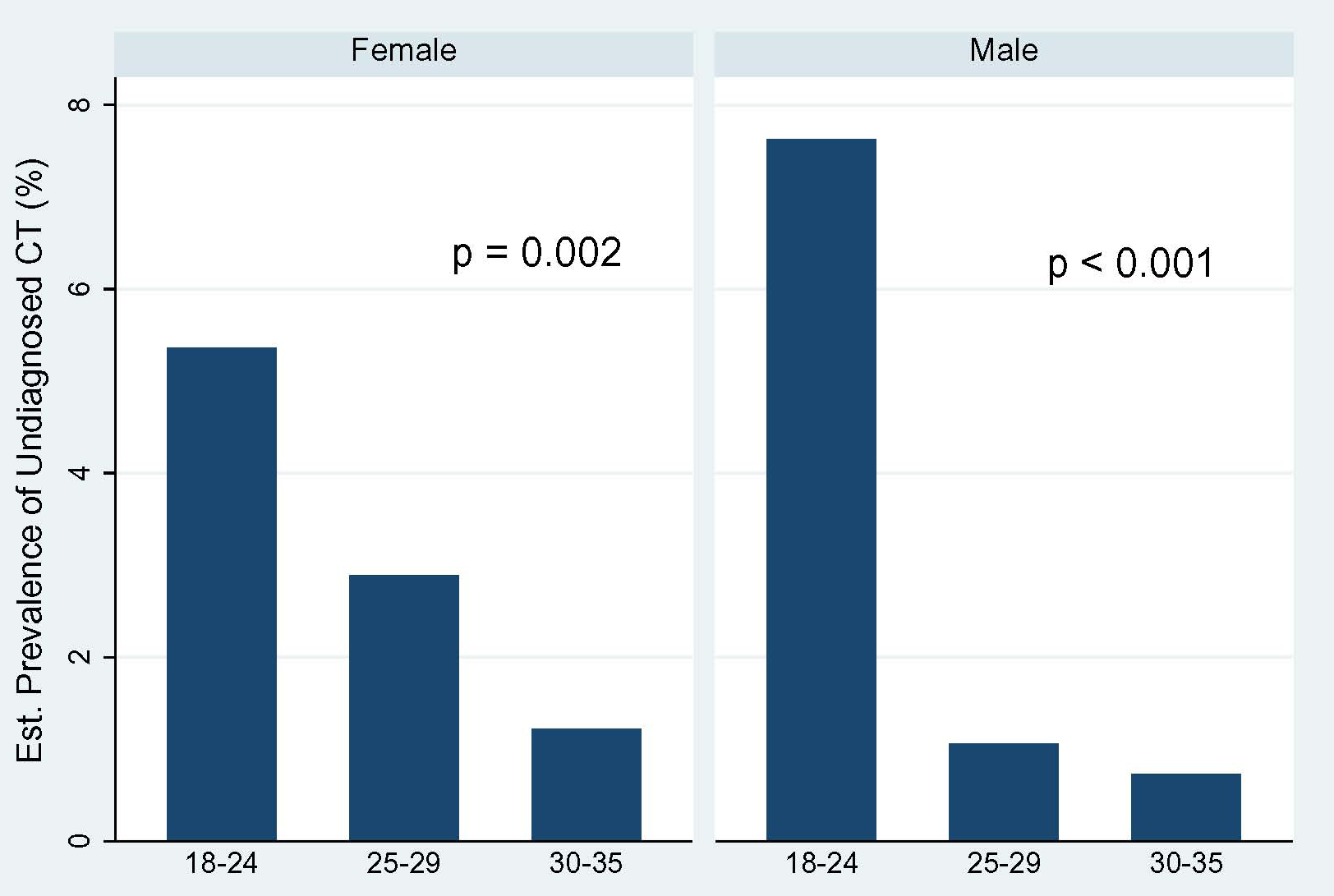

Supplement: Figure S1 — Estimated prevalence of undiagnosed chlamydial infections by age group and gender calcuated from combined 1997–98 and 2006–09 surveys. (TIF) [file pone.0089035.s001.tif]
