# Supplementary material for: Gender-Based Screening for Chlamydial Infection and Divergent Infection Trends in Men and Women
Source: PLoS One. 2014 Feb 19;9(2):e89035. doi: 10.1371/journal.pone.0089035 (PMC3929759; doi:10.1371/journal.pone.0089035)
Supplement: Table S1 — Counts of chlamydia cases among 18 to 35 year old adults reported to Baltimore City Health Department (BCHD) by gender and year, 1998 to 2009; together with estimated size of population ages 18 to 35 and calculated rate (Cases divided by Population). (DOCX) [file pone.0089035.s002.docx]

Table S1. Chlamydial infections reported to the Baltimore City Health Department by gender, 1998-2009.

|  | | | MALES | | | FEMALES | | |
| --- | --- | --- | --- | --- | --- | --- | --- | --- |
| YEAR | Cases | Population | | Rate (%) | Cases | | Population | Rate (%) |
| 1998 | 391 | 83,506 | | 0.47% | 3,255 | | 90,858 | 3.58% |
| 1999 | 372 | 83,539 | | 0.45% | 3,057 | | 91,735 | 3.33% |
| 2000 | 389 | 81,804 | | 0.48% | 3,156 | | 90,012 | 3.51% |
| 2001 | 374 | 80,375 | | 0.47% | 3,089 | | 88,745 | 3.48% |
| 2002 | 519 | 79,943 | | 0.65% | 3,562 | | 88,560 | 4.02% |
| 2003 | 570 | 80,152 | | 0.71% | 3,662 | | 88,372 | 4.14% |
| 2004 | 626 | 79,946 | | 0.78% | 3,713 | | 88,699 | 4.19% |
| 2005 | 660 | 80,777 | | 0.82% | 3,595 | | 89,354 | 4.02% |
| 2006 | 668 | 82,217 | | 0.81% | 3,601 | | 91,069 | 3.95% |
| 2007 | 861 | 83,984 | | 1.03% | 4,186 | | 92,781 | 4.51% |
| 2008 | 912 | 85,364 | | 1.07% | 4,918 | | 94,522 | 5.20% |
| 2009 | 956 | 86,648 | | 1.10% | 4,432 | | 96,489 | 4.59% |

Notes: Counts of chlamydia cases among 18 to 35 year old adults reported to Baltimore City Health Department (BCHD) by gender and year, 1998 to 2009, together with estimated size of population ages 18 to 35, and calculated rate (Cases divided by Population).

Population estimates derived from 1998 U.S. Census estimates and 1999-2009 Maryland Department of Planning (Maryland State Data Center), U.S. Census Bureau Intercensal Population Estimates published online at: (1) http://www.mdp.state.md.us/msdc/IntercensalEst00_10/IntercensalPopEstimates_MD_Jur_2000_2010.xls ; and (2) http://www.mdp.state.md.us/msdc/IntercensalEst90_99/A&R_md98.xls. To derive estimates of the size of the population ages 18 to 35, we assumed that: (1) 40% of population aged 15 to 19 were ages 18 and 19, and (2) 20% of the population aged 35 to 39 were age 35.
