# Supplementary material for: Gender-Based Screening for Chlamydial Infection and Divergent Infection Trends in Men and Women
Source: PLoS One. 2014 Feb 19;9(2):e89035. doi: 10.1371/journal.pone.0089035 (PMC3929759; doi:10.1371/journal.pone.0089035)
Supplement: Table S2 — Estimates for black respondents of gender-by-time (1997–98 vs. 2006–09) interaction using observed survey data on chlamydia prevalence plus imputations for missing data obtained by multiple imputation using chained equations (MICE) procedure. (DOCX) [file pone.0089035.s003.docx]

Table S2. Estimates of chlamydial infections for Black respondents by gender and time and interaction test results.

| Observed plus imputed (d) | Prevalence | SE | P (a) (Prevalence x Years) for Males & Females | P Interaction Test Gender x Time x Prevalence |
| --- | --- | --- | --- | --- |
| Black Males, 1997-98 | 1.6% | 1.6% |  |  |
| Black Males, 2006-09 | 7.2% | 1.8% | 0.049 |  |
| Black Females, 1997-98 | 6.1% | 2.1% |  |  |
| Black Females, 2006-09 | 4.6% | 0.9% | 0.485 (c) | 0.034 (b) |

Notes:

Estimates of chlamydial infections for Black respondents by gender and time (1997-98 vs. 2006-09)and of gender-by-time interaction using observed survey data on chlamydia prevalence plus imputations for missing data obtained by multiple imputation using chained equations (MICE) procedure. Imputations were performed using multiple imputation procedures of Stata v12 to impute the substantial number of missing chlamydial infection tests (n = 493 of 1,856 black respondents) plus the small number of missing education observations (n = 5), number of sex partners in past year (n = 2), married or not (n = 1), discharge (n = 2), gonorrhea diagnosis in past year (n = 8), chlamydia diagnosis in past year (n = 8). Other predictor variables used in imputations include age, male, time period (1997-98 vs. 2006-09), interaction of male-by-time period, dysuria, interaction of gender-by-time period, sample strata, and sample weight. (Note that reporting of dysuria and discharge were for past 2 months in 1997-98 BSBS and past 3 months in 2006-09 MSSP.) Logit models were used for all imputations except education and number of sex partners in past year. Imputation of these variables used ordered logit models. Multiple imputation procedure generated 60 sets of imputed data with burn-in period of 100 iterations.

(a) Logistic regression was used to test for trend over time (1997-98 vs. 2006-09) within the male and female subpopulations.

(b) Interaction test performed using logistic regression with controls for age, education, gender, number of sex partners in past year, married (or not), gender, year (1997-98 vs. 2006-09, and gender by year. Imputations and estimates were performed separately for Blacks.

(c) One stratum in the sample design was omitted because it contained no subpopulation members.

(d) Main effects for time period (1997-98 vs. 2006-09) and gender (male vs. female) were not statistically significant (Ps = 0.340 and > 0.50, respectively.) (Tested using logit model predicting CT as a function of time or gender.)
