# Supplementary material for: Gender-Based Screening for Chlamydial Infection and Divergent Infection Trends in Men and Women
Source: PLoS One. 2014 Feb 19;9(2):e89035. doi: 10.1371/journal.pone.0089035 (PMC3929759; doi:10.1371/journal.pone.0089035)
Supplement: Table S3 — Trends in estimated prevalence of undiagnosed chlamydial infections among Baltimore adults by gender and age group, 1997–98 and 2006–09. (DOCX) [file pone.0089035.s004.docx]

Table S3. Prevalence of chlamydial infections among Baltimore adults by gender and age group, 1997-98 and 2006-09

|  | 1997-98 | | | | 2006-09 | | | Time Trend 2006-09 vs. 1997-98 | |
| --- | --- | --- | --- | --- | --- | --- | --- | --- | --- |
| POPULATION | % | SE | 95% CI | % | | SE | 95% CI | OR | p |
| MEN |  |  |  |  | |  |  |  |  |
| Ages 18 – 24 | 2.1 | 1.2 | 0.69, 6.03 | 9.4 | | 2.4 | 5.56, 15.39 | 4.91 | 0.01 |
| Ages 25 – 29 | 2.9 | 1.6 | 0.93, 8.54 | 0.4 | | 0.4 | 0.06, 3.05 | 0.15 | 0.10 |
| Ages 30-35 | 0.0 | 0.0 | na | 1.0 | | 0.9 | 0.19, 5.15 | na | na |
| (p, across 3 age groups) [a] | (p = 0.17) | | | (p < 0.01) | | | |  |  |
| (p, by metric age) [b] | (p = 0.16) | | | (p < 0.01) | | | |  |  |
| WOMEN |  |  |  |  | |  |  |  |  |
| Ages 18 – 24 | 9.4 | 3.8 | 4.16, 19.86 | 4.4 | | 1.1 | 2.68, 7.07 | 0.44 | 0.11 |
| Ages 25 – 29 | 2.7 | 2.0 | 0.59, 11.24 | 3.0 | | 1.1 | 1.43, 6.051 | 1.12 | > 0.50 |
| Ages 30-35 | 0.8 | 0.8 | 0.11, 5.31 | 1.4 | | 0.7 | 0.55, 3.48 | 1.82 | > 0.50 |
| (p, across 3 age groups) [a] | (p = 0.01) | | | (p = 0.07) | | | |  |  |
| (p, by metric age) [b] | (p = 0.01) | | | (p = 0.01) | | | |  |  |

Notes: Results for adults ages 18 to 35 from the Baltimore STD and Behavior Survey (BSBS) and the Monitoring STIs Survey Program (MSSP). Table shows weighted estimates of the percentage of the population that was infected. Standard errors (SE) and 95% confidence intervals (CI) were calculated using statistical algorithms that take account of the complex sample designs of these surveys. Odds ratio (OR) contrast the estimated prevalence in 2006-09 to the estimated prevalence in 1997-98.

[a] Test for equivalence of prevalence estimates across the 3 age groups using design-based Pearson chi-square calculated by svy tab procedure of Stata v12.

[b] Test for equivalence of prevalence estimates by metric age using svy logit procedure of Stata v12.
