# Supplementary material for: Gender-Based Screening for Chlamydial Infection and Divergent Infection Trends in Men and Women
Source: PLoS One. 2014 Feb 19;9(2):e89035. doi: 10.1371/journal.pone.0089035 (PMC3929759; doi:10.1371/journal.pone.0089035)
Supplement: Text S1 — (DOC) [file pone.0089035.s005.doc]

**TEXT S1.**

**Study Design.** Estimates of trends in ***undiagnosed*** chlamydial infections were obtained from our two population surveys of probability samples of the Baltimore population in 1997-98 and 2006-09. These surveys collected biospecimens that were tested using NAAT assays to identify individuals with undiagnosed chlamydial infections. Since these surveys drew representative samples of the Baltimore population, estimates of the prevalence of undiagnosed chlamydial infection can be projected to the Baltimore population with known margins of sampling error.

**Survey Administration.** Respondents in the BSBS were randomized to complete a behavioral questionnaire in either an interviewer-administered computerized interview with some paper-and-pencil self-administered questions or in an audio computer-assisted self interview. Data from these two modes are aggregated for this analysis. The BSBS survey collected self-report data on sexual behavior, STI symptoms and history, alcohol and drug use, social attitudes and opinions, and demographic characteristics. The BSBS survey took on average 26 minutes to complete and respondents received $10 to $20.

In the MSSP, telephone interviewers from the University of Massachusetts at Boston screened and recruited eligible household members. Sample telephone numbers were randomly divided into replicates and released in a staggered manner to reflect a mix of newly released numbers, numbers that had been pursued for a short period of time and difficult to reach long term numbers. This strategy provided a cost effective manner of conducting the study while also giving maximum attention to pursuing every telephone number for the best outcome.

Minors aged 15-17 years in the MSSP were recruited with parental permission and minor assent. After describing the research project and obtaining consent, telephone interviewers transferred respondents to a T-ACASI system. The T-ACASI survey took 13 minutes on average to complete and collected information about participants’ sexual behavior, STI symptoms and history, and demographic characteristics. Respondents were compensated $10 to $20 for completing the survey. Further details on study design and survey administration for the MSSP are provided by Roman (2010).

**References**

Roman AM. Monitoring STIs Survey Program (MSSP): Methodological Report.
*Technical Papers on Health and Behavior Measurement,* No. 86. Washington DC: Program
on Health and Behavior Measurement, Research Triangle Institute, 2010. Available online at: <http://dragon.soc.Qc.cuny.edu/Staff/turner/TechPDFs/86_MSSP_Documentation.pdf>. Accessed January 2, 2012.
