# Supplementary material for: Gender-Based Screening for Chlamydial Infection and Divergent Infection Trends in Men and Women
Source: PLoS One. 2014 Feb 19;9(2):e89035. doi: 10.1371/journal.pone.0089035 (PMC3929759; doi:10.1371/journal.pone.0089035)
Supplement: Text S2 — (DOC) [file pone.0089035.s006.doc]

**TEXT S2.**

**Data Collection for Counts of Diagnosed Cases**. Counts of diagnosed chlamydial infection among 18 to 35 year olds (Fig 1a) reported to the Health Department were tabulated for calendar year 1998 (see Turner et al 2002) and for the field period of the 2006-09 MSSP (September 2006 through June 2009; tabulations by M Ruvva). Instances in which infection was reported in the same individual a second (or more) time within a 30-day period were excluded from these counts. For the survey period September 2006 through June 2009, reported case counts were summed and an annual average count (based on the 33-month survey period) was derived. Although the majority of reported cases (>85%) in any given period were Black, race was not recorded in up to 18% of women and 23% of men with reported diagnoses of chlamydial infection precluding tabulations of surveillance data by race/ethnicity. The estimated number of persons with undiagnosed infections (Fig 1b) was calculated as a product of the BSBS and MSSP survey estimates and U.S. Census estimates of the number of Baltimore residents aged 18 to 35 years. Population denominators for calculation of rates are: 83506 for men and 90858 for women in 1997-98 and 79842 for men and 87384 for women in 2006-09. (Ns are counts of Census Bureau population estimates for 1998 and the average for 2006 through 2009, <http://www.mdp.state.md.us/msdc//Pop_estimate/pop_Estimate.shtml>

Annual trend data are provided in Supplemental Table S1.

**References**

Turner CF, Rogers SM, Miller HG, et al. "Untreated gonococcal and chlamydial infection among a probability sample of adults." *JAMA*, 2002: 726-733.
