# Supplementary material for: Gender-Based Screening for Chlamydial Infection and Divergent Infection Trends in Men and Women
Source: PLoS One. 2014 Feb 19;9(2):e89035. doi: 10.1371/journal.pone.0089035 (PMC3929759; doi:10.1371/journal.pone.0089035)
Supplement: Text S3 — (DOC) [file pone.0089035.s007.doc]

**TEXT S3.**

**Diagnosed chlamydial infection.** During review of this article, it was suggested that readers might benefit from seeing the year-to-year fluctuations in the numbers of reported chlamydial cases in Baltimore. Table S1 provides that information for adults ages 18 to 35 by gender and year together with estimates of the size of the Baltimore population in that age range and the calculated case rates (Cases divided by Population).
