# Supplementary material for: Gender-Based Screening for Chlamydial Infection and Divergent Infection Trends in Men and Women
Source: PLoS One. 2014 Feb 19;9(2):e89035. doi: 10.1371/journal.pone.0089035 (PMC3929759; doi:10.1371/journal.pone.0089035)
Supplement: Text S4 — (DOC) [file pone.0089035.s008.doc]

**TEXT S4.**

**Landline telephone usage in Baltimore.** Over the course of the MSSP survey (September 2006 through June 2009), we estimate that approximately 15% of Baltimore households did not have a landline telephone. We derived this composite estimate using the National Health Interview Survey’s (NHIS) finding (Blumberg et al 2011) that the proportion of Baltimore Households that lacked a landline telephone was 14.1% in 2007, 15.2% in 2008, and 23.1% in 2009. (The NHIS is an in-person federal survey conducted for CDC by the Bureau of the Census with annual national samples of approximately 35,000 households; see www.cdc.gov/nchs/nhis/about_nhis.htm#sample_design.) For our composite estimate, we conservatively assumed that the unknown percent of Baltimore households without landline phone service in 2006 was as high as it was in 2007 (14.1%). We then calculated a weighted average adjusting for the relative number of specimens collected in each year of the survey.

**References**

Blumberg, SJ, et al*.* National Health Stat Report. *Wireless substitution: state-level estimates from the National Health Interview Survey, January 2007-June 2010.”,*2011. Apr 20; (39), 1-26, 28. National Center for Health Statistics, Hyattsville, MD.
