# Supplementary material for: Gender-Based Screening for Chlamydial Infection and Divergent Infection Trends in Men and Women
Source: PLoS One. 2014 Feb 19;9(2):e89035. doi: 10.1371/journal.pone.0089035 (PMC3929759; doi:10.1371/journal.pone.0089035)
Supplement: Text S5 — (DOC) [file pone.0089035.s009.doc]

**TEXT S5.**

**Sample weights.** Sample weights were derived for both surveys to adjust for the unequal probabilities of selection based on the sample design and for specimen nonresponse. An initial set of survey weights was developed as the inverse of the probability of selection to compensate for differences in selection probabilities across sample strata and within households with different numbers of eligible adults. Post-stratification adjustments were then applied to align the sample distributions with the 1998 and the 2006-08 U.S. Census estimates for the Baltimore City population by age, gender, and race/ethnicity. (We additionally adjusted the MSSP survey estimates by education.) A second set of poststratification weights was constructed to take account of subpopulation differences in the provision of biospecimens for STI testing among respondents who completed the survey interview.
