# Supplementary material for: Gender-Based Screening for Chlamydial Infection and Divergent Infection Trends in Men and Women
Source: PLoS One. 2014 Feb 19;9(2):e89035. doi: 10.1371/journal.pone.0089035 (PMC3929759; doi:10.1371/journal.pone.0089035)
Supplement: Text S6 — (DOC) [file pone.0089035.s010.doc]

**TEXT S6.**

**Biospecimen testing.** Following interview completion in both the BSBS and MSSP, survey respondents were asked to provide a biospecimen for *C. trachomatis* testing. Separate written consent was required for specimen testing. Respondents providing specimens were informed that they would be recontacted for a positive chlamydial test result and --- as required by Maryland law --- contact information for infected persons would be reported to the Baltimore City Health Department (BCHD).

In the BSBS survey, interviewers gave consenting respondents a urine collection kit which they used in privacy. Urine specimens were stored in chilled containers for delivery to the Johns Hopkins School of Medicine Chlamydia Lab. BSBS respondents providing urine specimens received an additional $10 to $20.

MSSP respondents who consented to provide a specimen were mailed a collection kit (a maximum of three days after the T-ACASI interview) with instructions, a consent form, and monetary compensation for completing the telephone survey. The vast majority of MSSP specimens were urine: 100% of male specimens and 96.5% of female specimens. A small number of women in 2009 (n =46) provided self-collected vaginal swabs. Urine specimens were collected in containers with DNA/RNA Protect^TM^ (Sierra Diagnostics, Sonora, CA), designed to prevent nucleic acid degradation for 7-10 days without refrigeration. Participants mailed their specimens in pre-addressed postage-paid shipping cartons to the University of North Carolina-Chapel Hill Hospitals’ McLendon Clinical Laboratories via U.S. Postal Service first class mail. Only specimens submitted with a signed consent form were tested. Participants received $40 to $100 for mailing in the specimen (payment for providing a specimen increased over the study period).
