# Supplementary material for: Gender-Based Screening for Chlamydial Infection and Divergent Infection Trends in Men and Women
Source: PLoS One. 2014 Feb 19;9(2):e89035. doi: 10.1371/journal.pone.0089035 (PMC3929759; doi:10.1371/journal.pone.0089035)
Supplement: Text S7 — (DOC) [file pone.0089035.s011.doc]

**TEXT S7.**

**Consent Procedures in MSSP.** Verbal consent was used for the telephone survey portion of the MSSP because there is no face-to-face contact in a telephone survey. Verbal consent is the widely-accepted norm for obtaining consent in telephone surveys. Household recruitment, screening, and all consent procedures were controlled by a computer-assisted, telephone interviewing (CATI) system. This system displayed the IRB-approved consent script for both adults and minors. Interviewers were required to enter key strokes indicating that respondents (and parent, if appropriate) had given verbal consent in order for the interview to proceed. Only participants who provided verbal consent were administered the telephone interview. Interview screening data, including the number of eligible persons in the household, the number of contacts, as well as parental refusal for minor participation were recorded by the CATI system.
