# Supplementary material for: Gender-Based Screening for Chlamydial Infection and Divergent Infection Trends in Men and Women
Source: PLoS One. 2014 Feb 19;9(2):e89035. doi: 10.1371/journal.pone.0089035 (PMC3929759; doi:10.1371/journal.pone.0089035)
Supplement: Text S8 — (DOC) [file pone.0089035.s012.doc]

**TEXT S8.**

**Imputation.** To assess the impact of missing biospecimens on our survey estimates of prevalence we employed both logistic prediction models and multiple imputation models using chained equations (MICE). MICE procedures were also used to impute the very small amount of missing sociodemographic data required for our substantive analyses.

**Single Imputation Modeling.** To assess the impact of biospecimen collection non-response on survey estimates of infection prevalence, we previously used logistic regression to model the likelihood that non-respondents would have tested positive based on sociodemographic, behavioral, and health-related variables. Our earlier BSBS analyses suggested that prevalence estimates were robust in the face of missing urine specimens (see Turner et al., 2002).

We subsequently carried out parallel analyses of the impact of missing biospecimens on MSSP estimates of infection prevalence. (A preliminary report of these analyses was presented in 2010 by Turner et al.) In the MSSP, a synthetic estimate of chlamydial prevalence was generated by combining: (1) results from 1,766 biospecimens provided by MSSP respondents, and (2) the imputed probability that the 705 respondents who did not provide specimens would have tested positive had they supplied a biospecimen. No infections were diagnosed among persons who reported zero lifetime partners and who did provide a biospecimen. We therefore imputed a zero probability of infection for the 66 persons who reported zero lifetime partners but who did *not* provide a biospecimen. Additional imputations were made using three logit models that predicted the probability of infection among the remaining 638 persons who did not provide a biospecimen but who reported one or more lifetime sexual partners. These models predicted the likelihood of infection as a function of: (1) respondents' sociodemographic characteristics, sexual behaviors, STI history, and reports of STI symptoms for the 616 biospecimen nonrespondents who had complete data for these predictors, (2) respondents sociodemographic characteristics and a reduced set of sexual behaviors and STI indicators for 11 biospecimen nonrespondents who had complete data only on this reduced set of predictors, and (3) respondent's sociodemographics for 11 biospecimen nonrespondents who did not have complete data on the predictor variables used in Model 2. The probability of infection derived using these predictive models was substituted for the missing observations. The synthetic estimate of chlamydia prevalence derived from these imputations for missing MSSP biospecimens was 3.58% which was nearly identical to the estimate (3.54%) derived from tested specimens alone.

**Multiple Imputation using Chained Equations (MICE).** MICE models were estimated to take account of the variance introduced by our imputations in testing our key substantive hypothesis. This modeling was restricted to the black population since there were few infections that could be used to estimate MICE models for the nonblack population (0 among nonblack females in 1997-98 and 3 in 2006-09; 4 among nonblack males in 1997-98 and 1 in 2006-09).

Our imputations used the MICE imputation procedures of Stata v12 (2011) to impute the substantial number of missing chlamydial infection tests (n = 493 of 1,856 black respondents) plus a small number of missing observations for education (n = 5), number of sex partners in past year (n = 2), marital status (married or not; n = 1), recent[[1]](#footnote-2) genital discharge (n = 2), gonorrhea diagnosis in past year (n = 8), and chlamydia diagnosis in past year (n = 8). Other predictor variables used in MICE imputations include age, male gender, time period (1997-98 vs. 2006-09), interaction of male-by-time period, interaction of gender-by-time period, sample strata, and sample weight. Logit models were used for all imputations except education and number of sex partners in past year. Imputation of these variables used ordered logit models. The chained equation multiple imputation procedure generated 60 sets of imputed data after a burn-in period of 100 iterations. These 60 MI replicate datasets were used to repeat the key statistical analyses we performed on cases with complete data and biospecimens. Our hypothesis testing using these MICE data took account of the complex sample designs of our population surveys using Stata’s *svy* estimation command.

Table S2 shows the estimated prevalence of undiagnosed infection in the population of young black adults in Baltimore over the time period 1997-98 to 2006-09. Using MICE-imputed data we conclude that there was a statistically significant (p = 0.049) increase in the estimated prevalence of undiagnosed chlamydial infection among black males from 1.6% (se = 1.6%) to 7.2% (se = 1.8%) while there was no significant change (p = 0.485) in estimated prevalence among black females. A test of the *gender by time period by prevalence* interaction (*p* = 0.034) suggests that trends over time in infection prevalence differed significantly for black males and black females.

**References**

StataCorp. *Multiple Imputation Reference Manual, Release 12.* College Station, TX: Stata Corporation; 2011.

Turner CF, Rogers SM, Miller HG, et al. "Untreated gonococcal and chlamydial infection among a probability sample of adults." *JAMA*, 2002: 726-733.

Turner CF, Rogers SM, and MSSP Research Team. Population Surveys of STIs: Bias due to Missing Biological Specimens. Abstract # 208792. American Public Health Asociation Meetings: Online Program, 2009. Available online at: [http://apha.confex.com/apha/137am/webprogram/Paper208792.html. Accessed January 2](http://apha.confex.com/apha/137am/webprogram/Paper208792.html.), 2012.

1. For a period of 3 months before survey in BSBS and 2 months in MSSP. [↑](#footnote-ref-2)
