# Supplementary material for: Gender-Based Screening for Chlamydial Infection and Divergent Infection Trends in Men and Women
Source: PLoS One. 2014 Feb 19;9(2):e89035. doi: 10.1371/journal.pone.0089035 (PMC3929759; doi:10.1371/journal.pone.0089035)
Supplement: Text S9 — (DOC) [file pone.0089035.s013.doc]

**TEXT S9.**

**Chlamydial Infections among Men who have Sex with Men (MSM).** During review of this article, a concern was raised about the possible impact on our results of trends in infection prevalence among men who had sex with men (MSM). A particular concern is that a spike in MSM infection prevalence in 2006-09 might account for our results for the male population. Further analyses of the MSSP data indicate that this is not the case.

Of 618 men who provided urine samples in 2006-09, 612 answered questions on the gender of their partners in the past 12 months. Of the 28 men reporting male sex partners, only one tested positive for chlamydia. Among men who reported no male partners, 20 of 584 tested positive for chlamydia. The weighted estimates of chlamydia prevalence in 2006-09 were 4.2% (CI: 2.5, 7.0) for the non-MSM sample and 1.6% (CI: 0.0, 11.7) for the MSM sample.
