# Supplementary material for: Gender-Based Screening for Chlamydial Infection and Divergent Infection Trends in Men and Women
Source: PLoS One. 2014 Feb 19;9(2):e89035. doi: 10.1371/journal.pone.0089035 (PMC3929759; doi:10.1371/journal.pone.0089035)
Supplement: Text S10 — (DOC) [file pone.0089035.s014.doc]

**TEXT S10.**

**Infection Prevalence and Age.** As noted in the article’s text, the estimated prevalence of undiagnosed chlamydial infection declines with age for both males and females; see Figure S1 which combines data from the 1997-98 and 2006-09 surveys. When data are disaggregated, we find that there is a significant association between metric age and the prevalence of undiagnosed infections for all groups --- except for males in 1997-98 (Table S3).

Because of small sample sizes, comparisons of trends over time in infection prevalence *within age groups* have limited statistical powers; see Table S3. Our analysis reveals that there are two borderline results that show declines over time in estimated prevalence. For females ages 18 to 24, the estimated prevalence declines from 9.4% (CI: 4.2, 19.9) to 4.4% (CI: 2.7, 7.1), but this result is of borderline significance (OR = 0.4, *p* =0.11). For males ages 25 to 29, the estimated prevalence declines from 2.9% (CI: 0.9, 8.5) to 0.4% (CI: 0.1%, 3.1%), but this result is also of borderline significance (OR = 0.2, *p* = 0.10).

The exception to this pattern occurs for males ages 18 to 24. The estimated prevalence of undiagnosed infection in this group rises from 2.1% (CI: 0.7, 6.0) in 1997-98 to 9.4% (CI: 5.6, 15.4) in 2006-09. This increase (OR = 4.9) in the prevalence of undiagnosed infections is statistically significant (*p* = 0.01).
