# Supplementary material for: Gender-Based Screening for Chlamydial Infection and Divergent Infection Trends in Men and Women
Source: PLoS One. 2014 Feb 19;9(2):e89035. doi: 10.1371/journal.pone.0089035 (PMC3929759; doi:10.1371/journal.pone.0089035)
Supplement: Text S11 — (DOC) [file pone.0089035.s015.doc]

**TEXT S11.**

For example, Gindi et al. (2011) found that 44% of males and 50% of females formed sexual relationships with partners ***who lived in the same or adjacent Census tracts.*** (Data from the 2007 cycle of the National HIV Behavioral Surveillance System Surveys.)

**References**

[Gindi RM](http://www.ncbi.nlm.nih.gov/pubmed?term=Gindi RM%5BAuthor%5D&cauthor=true&cauthor_uid=20966827), [Sifakis F](http://www.ncbi.nlm.nih.gov/pubmed?term=Sifakis F%5BAuthor%5D&cauthor=true&cauthor_uid=20966827), [Sherman SG](http://www.ncbi.nlm.nih.gov/pubmed?term=Sherman SG%5BAuthor%5D&cauthor=true&cauthor_uid=20966827), [Towe VL](http://www.ncbi.nlm.nih.gov/pubmed?term=Towe VL%5BAuthor%5D&cauthor=true&cauthor_uid=20966827) et al (2011). The geography of heterosexual partnerships in Baltimore city adults. [*Sex Transm Dis.*](http://www.ncbi.nlm.nih.gov/pubmed/20966827) Apr;38(4):260-6. doi: 10.1097.
