# Supplementary material for: Gender-Based Screening for Chlamydial Infection and Divergent Infection Trends in Men and Women
Source: PLoS One. 2014 Feb 19;9(2):e89035. doi: 10.1371/journal.pone.0089035 (PMC3929759; doi:10.1371/journal.pone.0089035)
Supplement: Text S12 — (DOC) [file pone.0089035.s016.doc]

**TEXT S12.**

In 2010, the average rate of chlamydia cases nationally 426 per 100,000 of population while this rate was 1,293 per 100,000 in Baltimore (CDC 2010)

**References**

Centers for Disease Control (2010). Sexually Transmitted Disease Surveillance, 2010, page 7 and Table 9.
