# Supplementary material for: Correction: Gender-Based Screening for Chlamydial Infection and Divergent Infection Trends in Men and Women
Source: PLoS One. 2014 May 28;9(5):e99374. doi: 10.1371/journal.pone.0099374 (PMC4037223; doi:10.1371/journal.pone.0099374)
Supplement: Text S13 — (DOCX) [file pone.0099374.s001.docx]

**TEXT S13**

It should be noted that the sample design for the 1997-98 BSBS drew a probability sample of properties from the Baltimore Real Estate Property Registry. Interviews and urine samples were obtained from 471 randomly selected respondents residing in 471 households that contained a single dwelling unit and from 108 respondents residing in 108 households at 34 multi-unit properties. (For these multi-unit properties, the median number of households included in the completed sample was 3 per property with a range of 2 to 7). The clustering of households within these multi-unit properties has a trivial effect on the variance of our estimates of the prevalence of undiagnosed chlamydial infections. Our 1997-98 chlamydia prevalence estimate of 3.0% has a standard error of 0.843% when this clustering is ignored and 0.841% when it is accounted for. The analyses presented in this article do not model the impact of this multi-unit clustering and thus our analyses are trivially biased against finding a significant trend over time. (The 2006-09 MSSP sample design did not involve any clustering of sample households.)
